# Aquaponics-Derived Tilapia Skin Collagen for Biomaterials Development

**DOI:** 10.3390/polym14091865

**Published:** 2022-05-02

**Authors:** Nunzia Gallo, Maria Lucia Natali, Alessandra Quarta, Antonio Gaballo, Alberta Terzi, Teresa Sibillano, Cinzia Giannini, Giuseppe Egidio De Benedetto, Paola Lunetti, Loredana Capobianco, Federica Stella Blasi, Alessandro Sicuro, Angelo Corallo, Alessandro Sannino, Luca Salvatore

**Affiliations:** 1 Department of Engineering for Innovation, University of Salento, Via Monteroni, 73100 Lecce, Italy; marialucia.natali@unisalento.it (M.L.N.); federicastella.blasi@unisalento.it (F.S.B.); angelo.corallo@unisalento.it (A.C.); alessandro.sannino@unisalento.it (A.S.); luca.salvatore@unisalento.it (L.S.); 2 Typeone Biomaterials Srl, Via Vittorio Veneto, 73036 Lecce, Italy; 3 CNR Nanotec, Institute of Nanotechnology, Via Monteroni, 73100 Lecce, Italy; alessandra.quarta@nanotec.cnr.it (A.Q.); antonio.gaballo@nanotec.cnr.it (A.G.); 4 Institute of Crystallography, National Research Council, 70125 Bari, Italy; alberta.terzi@ic.cnr.it (A.T.); teresa.sibillano@ic.cnr.it (T.S.); cinzia.giannini@ic.cnr.it (C.G.); 5 Department of Cultural Heritage, University of Salento, Via Monteroni, 73100 Lecce, Italy; giuseppe.debenedetto@unisalento.it; 6 Department of Biological and Environmental Sciences and Technologies, University of Salento, Via Monteroni, 73100 Lecce, Italy; paola.lunetti@unisalento.it (P.L.); loredana.capobianco@unisalento.it (L.C.); alessandro.sicuro@unisalento.it (A.S.)

**Keywords:** type I collagen, tilapia, skin, aquaponic, biomaterials

## Abstract

Collagen is one of the most widely used biomaterials in health-related sectors. The industrial production of collagen mostly relies on its extraction from mammals, but several issues limited its use. In the last two decades, marine organisms attracted interest as safe, abundant, and alternative source for collagen extraction. In particular, the possibility to valorize the huge quantity of fish industry waste and byproducts as collagen source reinforced perception of fish collagen as eco-friendlier and particularly attractive in terms of profitability and cost-effectiveness. Especially fish byproducts from eco-sustainable aquaponics production allow for fish biomass with additional added value and controlled properties over time. Among fish species, *Oreochromis niloticus* is one of the most widely bred fish in large-scale aquaculture and aquaponics systems. In this work, type I collagen was extracted from aquaponics-raised Tilapia skin and characterized from a chemical, physical, mechanical, and biological point of view in comparison with a commercially available analog. Performed analysis confirmed that the proprietary process optimized for type I collagen extraction allowed to isolate pure native collagen and to preserve its native conformational structure. Preliminary cellular studies performed with mouse fibroblasts indicated its optimal biocompatibility. All data confirmed the eligibility of the extracted Tilapia-derived native type I collagen as a biomaterial for healthcare applications.

## 1. Introduction

Type I collagen is the predominant structural component of vertebrates’ connective tissues that accounts for approximately 70% of the total collagens found in the human body [1,2]. Being one of the major extracellular matrix (ECM) components, it is intrinsically bioactive, biodegradable, and particularly low immunogenic and weak antigenic [2,3,4,5,6,7]. Therefore, its employment as a biomaterial in the food, pharmaceutical, cosmetic, and biomedical industries is not surprising. Particularly high is its request in the health-related sector that makes extensive use of type I collagen for the manufacture of several kinds of formulations for tissue restoration/regeneration [2].

Collagen used in the biomedical field is usually derived from animal tissues. Large terrestrial mammals (i.e., bovine, porcine, equine, ovine) are currently the preferred sources for collagen extraction, for the high sequence homology with human collagen (>90%) [3] as well as for the possibility of accessing large quantities of raw materials. However, the application potential of mammalian-derived collagen is limited by issues such as triggering immune reactions (about 3% of the population), zoonosis transmission risks (i.e., the foot and mouth disease and the group of the bovine spongiform encephalopathies), besides cultural and religious concerns [3,8,9].

In this perspective, in the last two decades, marine organisms have attracted interest as safe, alternative, and abundant sources for collagen extraction [10,11,12]. Apart from owing biocompatibility, bioactivity, and biodegradability, fish collagen revealed an intrinsically lower threat of transmissible diseases, freedom from religious concerns [9], and weak antigenicity [13]. Marine collagen and its derivates (i.e., gelatin) were revealed to be easily processed in different types of formulations (i.e., injectable hydrogels, implantable temporary scaffolds, orally administrable pills) and relative properties tunable to suit diverse applications in a variety of biomedical fields [14,15,16,17,18].

Moreover, the possibility of developing waste recovery technologies to obtain high added value products from the abundant discards (e.g., skin, bones, fins, heads, guts, and scales) of the fish processing industry (70–85% of the total weight of catch) is of large scientific and industrial interest [10,19,20]. In particular, Nile Tilapia (*Oreochromis niloticus*), one of the World’s most representative species of the fisheries and aquaculture food sector, attracted interest as a byproduct source for collagen extraction. Tilapia skin was demonstrated to provide for 28–40% dry weight yield of acid-soluble collagen (ASC) or pepsin soluble collagen (PSC) [21,22,23,24]. The fast growth speed, adaptability to a wide range of environmental conditions, ability to grow and reproduce in captivity, easy feeding at low trophic level, and easy processing to fish fillets [25] made Tilapia the second most important group of farmed fish after carps [22,26], with a global production of 6.5 million tons in 2018 and an aquaculture production increasing 11% per year [27]. The possibility to tune growth conditions and produce hazardous-free commercial products with controlled and reproducible final properties through, e.g., the aquaponics farming method, gives to Tilapia fillets and byproducts a high added value. Aquaponics is, in fact, known as a form of sustainable aquaculture, because it imitates natural systems, where aquatic organisms and plants grow symbiotically (the latter using nutrients from the fish waste processed by nitrifying bacteria) [28]. As a result, it proves to have higher water use efficiency than conventional aquaculture and agriculture, it does not use pesticides, and even the use of fertilizers is reduced, which makes aquaponics greener and more sustainable than conventional techniques [28]. For this reason, the valorization of aquaponics Tilapia-waste polluting byproducts as sources of collagen makes the derived biomaterial not only eco-friendlier but also particularly attractive in terms of profitability and cost-effectiveness [29].

In this study, the physical, chemical, and biological properties of a fibrillar type I collagen isolated from an aquaponics-derived Tilapia skin were extensively assessed and compared with those of a commercially available isoform from the same species and tissue. The identity and the nativeness of the extracted protein were assessed by Poly-Acrylamide Gel Electrophoresis in the presence of Sodium Dodecyl Sulphate (SDS-PAGE). The collagen secondary structure was investigated by Fourier Transform Infrared Spectroscopy (FT-IR), while its ultrastructure by Wide-angle X-ray scattering (WAXS). The amino acid composition was investigated by Gas Chromatography coupled with Mass Spectrometry (GC-MS). The thermal behavior was determined by Differential Scanning Calorimetry (DSC). Static contact angle measurements were conducted to achieve information about the surface hydrophobic character. The collagen mechanical response was assessed by the uniaxial tensile test. Lastly, the cytocompatibility was assessed by two standard assays, 3-[4,5-dimethylthiazol-2-yl]-2,5 diphenyl tetrazolium bromide (MTT) test and Live/Dead. The viability, the morphology, and the distribution of 3T3 mouse fibroblasts seeded over collagen films were followed up for 12 days.

## 2. Materials

Type I collagen (T) was extracted from the skin of Nile Tilapia (*Oreochromis niloticus*) bred in the pilot aquaponics plant inside the Dept. of Innovation Engineering’s Urban Farming Lab of the (University of Salento, Lecce, Italy). This plant consists of 3 cooperating subsystems (the recirculating aquaculture systems, the biofilter, and the hydroponic cultivation system), which ensure that the nutrients contained in the fish feed and feces are used to grow plants without wasting water. Both the fish biomass and the environmental parameters are constantly checked through an innovative ICT monitoring, control, and implementation system, based on IoT (Internet of Things) technologies, ensuring that the whole aquaponics system is managed efficiently. Nile Tilapia was fed tailored commercial feed EFICO Cromis 832F 3 (BioMar SAS, Nersac, France). Tilapia specimens of about 16–17 months with a mean weight of about 301 ± 48 g, 25.4 ± 1.3 × 8.4 ± 0.5 cm (l × h) sized, were selected for collagen extraction.

Collagen extraction was performed according to a proprietary process developed by Typeone Biomaterials Srl (Lecce, Italy) and provided in dry flakes. An analogous suspension of a commercial type I collagen (N) from Tilapia skin was provided in dry flakes from Nippi Inc. (Tokyo, Japan) and used for comparative analysis. Aqueous suspensions (10 mg/mL) were obtained by slowly hydrating collagen dry flakes in acetic acid 0.2 M for 3 h under magnetic stirring at 4°C in order to avoid collagen denaturation. Distilled water was obtained from the Millipore Milli-U10 water purification facility from Merck KGaA (Darmstadt, Germany). Standard proteins for SDS-PAGE of precise molecular weights ranging from 10 to 250 kDa were provided by Bio-Rad Laboratories Inc (Hercules, CA, USA). N, N-dimethylformamide was provided by VWR International PBI S.r.l. (Milan, Italy). Acetic acid, norleucine, and *N*-tert-butyldimethylsilyl-*N*-methyltrifluoroacetamide (MTBSTFA) were purchased by Sigma-Aldrich (Milan, Italy). For the cellular assays, culture media, supplements, trypsin and MTT were purchased from Sigma-Aldrich (Milan, Italy). The Live/Dead assay was purchased from Thermo Fisher Scientific Inc. (Waltham, MA, USA). If not otherwise stated, all other chemicals used were of analytical grade and purchased by Sigma-Aldrich (Milan, Italy).

## 3. Methods

### 3.1. Extracts Purity and Integrity

T and N purity and molecular weight were firstly assessed by SDS-PAGE using a Mini-Protean Tetra Cell system from Bio-Rad Laboratories, Inc. (Hercules, CA, USA). Hand cast gels (5% stacking gel, 7% resolving gel) were prepared using acrylamide/bisacrylamide solution with a ratio of 37.5:1. About 0.3 g of T and N collagen gels (10 mg/mL) were dissolved in 0.5 mL of reducing solution (Urea 2 M, Laemmli buffer: 62.5 mM Tris-HCl pH 6.8, 10% glycerol, 2% SDS, 0.01% bromophenol blue, 5% β-mercaptoethanol) and heated at 50 °C for 1 h [30,31,32,33,34]. Native type I collagen from horse tendon was also subjected to the reducing treatment to provide an example of protein integrity and purity [33,34]. After 1 min at max speed centrifugation, a few μL of supernatants were withdrawn and subjected to the electrophoretic run at 70 V for about 10 min in the stacking gel and at 120 V for about 2 h within the resolving gel in the presence of protein standards of precise molecular weights ranging from 10 to 250 kDa. At the end of the run, the gel was rapid Coomassie stained and acquired [34,35]. Then, the revealed protein bands were analyzed by mean of GelAnalyzer 19.1 (www.gelanalyzer.com, accessed on the 4 November 2021) by Istvan Lazar Jr., PhD and Istvan Lazar Sr., PhD, CSc for protein subunits ratio and molecular weight determination.

### 3.2. Amino Acid Composition

The amino acid composition of T and C was investigated by mean of GC-MS as described elsewhere [36,37], with slight modifications. Briefly, after hydrolysis in 6 N hydrochloric acid for 2 h at 120 °C, samples were transferred into a glass vial; norleucine was added as an internal standard (5 μL of a 100 ng/μL norleucine solution) and freeze-dried [36,37]. After lyophilization, the residues were reconstituted with 70 μL of *N*, *N*-dimethylformamide, and 20 μL of MTBSTFA. Derivatization with MTBSTFA was performed at 100 °C for 60 min. After cooling the solution at room temperature for 5 min, 1 μL of the solutions was injected in spit mode (split ratio 10:1). Samples were run on a GC-QqQ-MS (Bruker 456 gas chromatograph coupled to a triple quadrupole mass spectrometer Bruker Scion TQ) equipped with an autosampler (GC PAL, CTC Analytics AG). The GC was operated at a constant flow of 1.0 mL/min, and analytes were separated on an HP 5 MS capillary column (50 m with a 2 m guard column, inner diameter 250 μm, and film thickness 0.25 μm). The oven was kept at 60 °C for 1 min after injection, then a temperature gradient of 10 °C/min was employed until 320 °C was reached. The oven was then held at 320 °C for 10 min. The total run time was 37 min. The mass detector was operated at 70 eV in the electron impact (EI) ionization mode scanning the mass range 50–550 Da. The ion source and transfer line temperatures were 230 °C and 280 °C, respectively. Bruker MS Workstation 8.2 software was used to acquire chromatograms, process the data, and quantify amino acids. Samples were run thrice.

### 3.3. Structural Analysis

FT-IR was performed by means of FTIR-6300 from Jasco GmbH (Pfungstadt, Germany) in order to investigate the protein identity and the triple helical structure integrity. Briefly, T and N aqueous suspensions (5 mg/mL) were under vacuum degassed, casted into Petri dishes and air-dried for 72 h in a laminar flow hood. Films were then 1 × 1 cm cut and placed in the reading area. Absorption spectra were recorded in the range 1800–800 cm^−1^ at a resolution of 4 cm^−1^, smoothed according to the Savitsky–Golay method, and analyzed by mean of the Origin software from OriginLab Corporation (Northampton, MA, USA) [34]. Peak positions were designated according to the spectrum shapes to make sure that all the wavelength ranges of the β-sheet (1610–1642 cm^−1^), random coil (1642–1650 cm^−1^), α-helix (1650–1660 cm^−1^), β-turn (1660–1680 cm^−1^), and β-antiparallel (1680–1700 cm^−1^) had the designated peak positions. Then, the area of the deconvoluted secondary structures detected were calculated. The secondary structures percentage was calculated by dividing the peak area of each secondary structure by the whole peak area of all the secondary structures [38]. Additionally, the relative number of triple helices (ca. 1630 cm^−1^) with respect to α-helices was calculated as the percentage of the total amide I peak area ascribable to the triple helix peak [33,39]. Four samples for each sample type were scanned, and each spectrum was collected as the average of 64 scans.

### 3.4. X-rays Structural Analysis

WAXS experiments were performed on T and N at the X-ray Micro Imaging Laboratory (XMI-LAB) [40], which is equipped with a Fr-E+ SuperBright copper anode MicroSource (λ = 0.154 nm, 2475 W) coupled through a focusing multilayer optics ConfocalMax-Flux (CMF 15–105) to a 3-pinholes camera for X-ray microscopy. The beam size was about 0.5 × 0.5 mm^2^. In order to have access to a range of scattering vector moduli (q = 4 psinϑ/λ) from 0.3 to 3.5 Å^−1^, corresponding to a 1.8 ÷ 21 Å range in the direct space, an Image Plate (IP) detector (250 × 160 mm^2^, 100μm effective pixel size) placed at ∼10 cm distance from the sample was employed for WAXS data collection. All measurements were digitally transformed by an off-line RAXIA reader. The data were elaborated by SAXSGUI and SUNBIM software [41]. All the samples were placed in the Ultralene^®^ sachet for the measurement.

### 3.5. Thermal Properties

DSC allows measuring protein thermal behavior along with their denaturation temperature (T_d_). Thermograms of T and N were determined using a Q2000 Series DSC from TA Instruments (New Castle, DE, USA). T and N gels were accurately weighed (5–10 mg) into aluminum pans, hermetically sealed, and scanned from 5 °C to 80 °C at 5 °C/min in an inert nitrogen atmosphere (50 mL min^−1^) [34,42]. An empty aluminum pan was used as a reference probe. The T_d_ was measured as the mid-point of the corresponding endothermic peak [33,34,43]. The area under the peak allowed for estimating the enthalpy required for the transition [33,43]. Each sample was run in triplicate.

### 3.6. Wettability

Static contact angle measurements were performed by dropping 10 μL milli-Q water on 10 × 10 mm T and C films using the sessile-drop method using a FTA 1000 analysis system (First Ten Angstroms, Newark, NJ, USA) [34]. An average of three drops was conducted for each sample type.

### 3.7. Mechanical Properties

The constitutive bond of Tilapia skin-derived collagens was evaluated in a hydrated state using a ZwickiLine universal testing machine (Zwick/Roell, Ulm, Germany) equipped with a loading cell of 1 kN. Samples of T and N (5 × 20 mm) were hydrated in 0.01 M PBS at room temperature for 1 h, clamped and tensile tested under displacement-control until failure with a preload of 0.1 N and a load speed of 0.1 mm/s [34]. The Young modulus (E), the stress at break (σmax), and the strain at break (𝜀r) were measured. In particular, E was calculated as the slope of the linear elastic region of the stress–strain curve at low strain values (in the range 1–5%). The thickness and width of wet specimens were measured using a Dino-Lite digital microscope (AnMo Electronics Corporation, New Taipei City, Taiwan). The experiment was performed in triplicate for each sample type.

### 3.8. Biocompatibility

Mouse fibroblasts, namely 3T3, were purchased from ATCC (Manassas, VA, USA) and cultured in high glucose Dulbecco’s Modified Eagle Medium (DMEM) supplemented with 10% Fetal Bovine Serum (FBS), 100 U mL^−1^ penicillin and 100 μg mL^−1^ streptomycin at 37 °C in an atmosphere of 5% CO_2_. To assess the cytocompatibility of T and N, fibroblasts were seeded into cell culture plates previously coated with T or N gels. In brief, prior to cell seeding, T and N gels (0.3 mL with a concentration equal to 5 mg/mL) were casted at the bottom of 24-well culture plates and let dry for 72 h; upon casting, plates were exposed to UV light irradiation for 2 h and then equilibrated overnight in cell culture medium at 37 °C. Then, 2 × 10^4^ cells in 1 mL of culture medium were seeded into each well. Cells seeded directly into the multiwells were used as control samples. At 3, 6, and 12 days after seeding the medium was removed from the plates, and the samples were washed twice with phosphate buffer saline (PBS) prior to proceeding with the assays. Two kinds of tests were performed, the Live/Dead assay kit and the standard MTT assay.

In the case of the Live/Dead assay, a PBS solution containing calcein and ethidium homodimer was prepared according to the manufacturer’s indications, added to the cell culture plated, and incubated at 37 °C for 1 h. The activity of intracellular esterase induces non-fluorescent, cell-permeant calcein acetoxymethyl ester to become fluorescent after hydrolysis, giving a green fluorescence to the viable cells. Conversely, ethidium homodimer enters only into damaged cells and binds to nucleic acids producing a red fluorescence that indicates dead cells. Finally, the solution was replaced with fresh PBS before imaging under the Fluorescence Microscope EVOS FLoid Cell Imaging Station (ThermoFisher, Waltham, MA, USA). To obtain a quantitative estimation of cell viability, the fluorescent pixels of both the green channel (live cells) and the red channel (dead cells) of 3 independent images for each type of sample were quantified with ImageJ Software (Rasband, W.S., ImageJ, U. S. National Institutes of Health, Bethesda, MD, USA) and averaged. Then, the percentage of living cells over the number of total cells was estimated as follows:

$$\mathrm{Viability}\;\left(\%\right)=\frac{\mathrm{Average}\;\mathrm{pixels}\;\mathrm{of}\;\mathrm{the}\;\mathrm{green}\;\mathrm{channel}\;}{\left(\mathrm{Average}\;\mathrm{pixels}\;\mathrm{of}\;\mathrm{the}\;\mathrm{green}\;\mathrm{channel}+\mathrm{Average}\;\mathrm{pixels}\;\mathrm{of}\;\mathrm{the}\;\mathrm{red}\;\mathrm{channel}\right)}*100$$

To perform the MTT assay, at 3, 6, and 12 days after cell seeding, MTT was dissolved into a culture medium without serum (final concentration 500 µg/mL). Then, 1 mL of the medium was added to each plate and incubated for 2 h at 37 °C. Subsequently, it was removed, and the dark-blue formazan crystals produced by MTT metabolization were solubilized by dimethyl sulfoxide. Finally, the absorbance of the obtained solutions was measured using the CLARIO star Plus microplate reader (BMG Labtech, Ortenberg, Germany), (*λ =* 570 nm) and was considered proportional to cell proliferation. The assay was also performed on blank film samples (i.e., T and N gels without cells) to assess colorimetric interference by the gels themselves. For each sample, an average value derived from *n* = 3 independent replicates were calculated and expressed as a percentage of viable cells over control cells (i.e., cells seeded on the standard well surface and considered as 100% viable).

The percentage of cell viability was determined according to the following formula:

$$\mathrm{Viability}\;\left(\%\right)=\frac{\mathrm{Absorption}\;\mathrm{of}\;\mathrm{the}\;\mathrm{treated}\;\mathrm{sample}}{\mathrm{Absorption}\;\mathrm{of}\;\mathrm{the}\;\mathrm{control}\;\mathrm{sample}}*100$$

### 3.9. Statistical Analysis

All data were expressed as mean ± the standard deviation. Statistical significance of experimental data was determined using *t*-Student test. Differences were considered significant at *p* < 0.05. 

## 4. Results

### 4.1. Extracts Purity and Integrity

**Figure 1:**
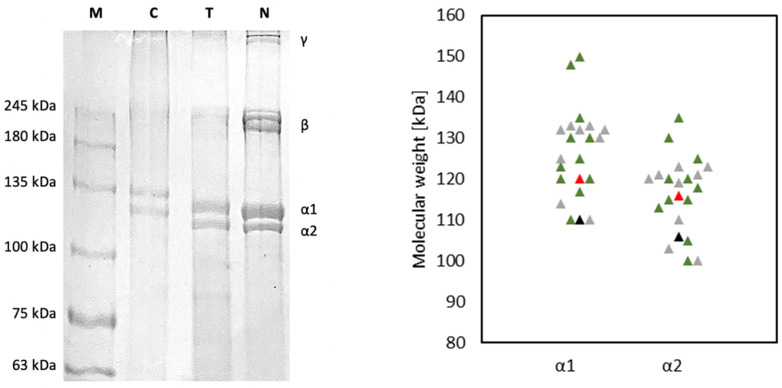
On the **left**: Comparison of electrophoretic pattern of T and N; proteins were separated by SDS-PAGE and Coomassie stained; high molecular weight protein markers (MW) ranging from 10 to 250 kDa were used to estimate the molecular weight of the proteins, type I collagen from horse tendon (C) was used as an example of integrity and purity. On the **right**: comparison of the molecular weight of α1 and α2 chains of T (red) and N (black) with literature data about α1 and α2 of ASC (green) and PSC (grey) isolated from Tilapia skin [23,38,44,45,46,49,50,51,52,53,54,55,57].

The electrophoretic patterns of T and N are shown in Figure 1 and were characterized by protein bands attributable to the two type I collagen α1 and α2 chains of about 120 kDa and 110 kDa respectively, as reported in the literature for collagen obtained from Tilapia skin [23,38,44,45,46,47,48,49,50,51,52,53]. In particular the α1 chain was found at 120 ± 3 kDa for T and 116 ± 2 kDa for N, while α2 chain was found at about 110 ± 2 kDa for T and 106 ± 1 kDa for N (α1, *p* = 0.02; α2, *p* = 0.03). High molecular weight components, including γ chains (trimers) and β chains (dimers) were present. In particular, β chains were found at about 242 ± 12 kDa for T and at 226 ± 8 kDa for N (γ, *p* = 0.03) [23,45,46,52,54,55,56]. Furthermore, no non-collagenous protein bands were observed, indicating the purity of both collagens and the preservation of their native structure after the extraction process. As expected, the band intensity of α1 was higher than that of α2 by approximately 2-fold, confirming that the extracted collagens contained two identical subunits of α1 and only one α2, which was consistent with the molecular composition of type I collagen (α1)_2_α2. The band intensity ratio of cross-linked chains (β + γ) to non-cross-linked monomer chains (α1 + α2) was calculated to evaluate the efficacy of the extraction method in disassembling fibril units [23]. The (β + γ)/(α1 + α2) ratio was found to be 0.28 ± 0.11 for T and 0.77 ± 0.16 for N (*p* = 0.002). In T more β and γ chains were converted to monomer chains (α1 and α2), suggesting how the extraction process optimized for T was more effective in collagen fibrils disassembly [23].

### 4.2. Amino Acid Composition

**Figure 2:**
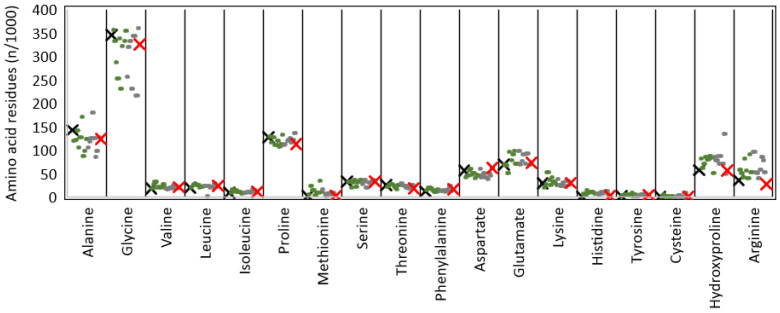
Comparison of the amino acid relative abundance of T (red) and N (black) with the literature data from ASC (green) and PSC (grey) isolated from Tilapia skin [22,23,24,44,45,50,51,52,54,57,58,59].

The amino acid composition of T, expressed as number of residues per 1000 total amino acid residues, was reported in Table 1 and was found to be comparable to N. Moreover, it was found to be in line with literature data on Tilapia skin-derived collagen (Figure 2) [22,23,24,44,45,50,51,52,54,57,58,59], common aquatic organisms [9], and very similar to land mammals [3]. Collagens are typically characterized by a repetitive tripeptide (Glycine-X-Y)n structure, where X and Y positions are usually occupied by a residue of proline and hydroxyproline, respectively. As expected, both T and N samples were found to be rich in glycine, proline, alanine, and hydroxyproline. Glycine, which accounts for about one-third of the total residues, is the most abundant species with 325 and 345 residues in T and N, respectively. The X position of the (Glycine-X-Y)n repetition is often occupied by proline, which is consistent with results reported in Table 1 (112 and 127 proline residues in T and N). The Y position is usually occupied by hydroxyproline, with a content of about 56 residues for both. The hydroxyproline content, although it was found to be lower than the literature data, is known to be correlated with living conditions [60] and feeding. However, to the best of our knowledge, it is not possible to correlate with certainty the low hydroxyproline content to specific species features and breeding conditions. The imino acid content of T and N was found to be about 168 and 184 residues/1000 amino acid residues for T and N, respectively. The higher imino acid content of N reflected its greater structural rigidity since a higher pyrrolidine rings content imposes more restrictions on the polypeptide chain conformation.

### 4.3. Structural Analysis

**Figure 3:**
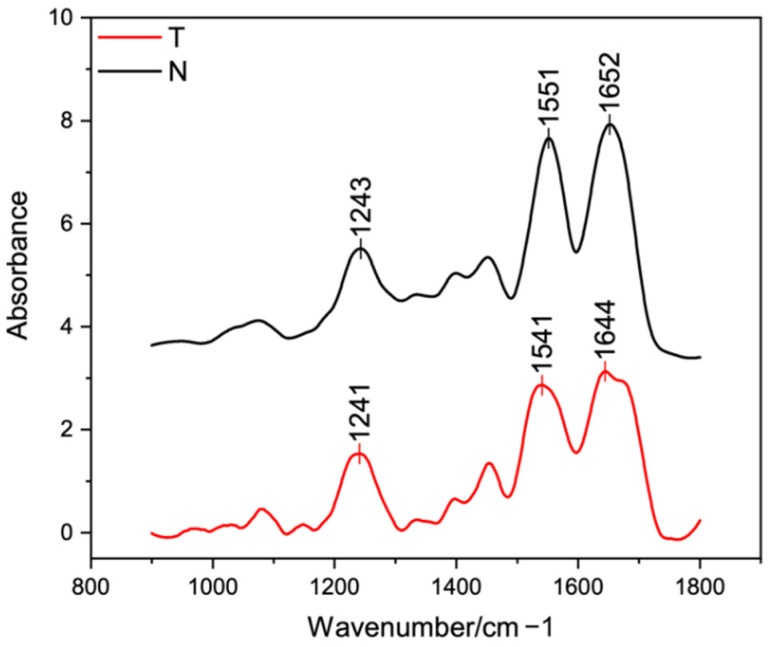
FT-IR spectra of T (red) and N (black) samples (A).

FT-IR spectra of T and N were both characterized by the presence of the three typical peaks of type I collagen (Figure 3) from land animals [32,33,61,62] and from Tilapia skin [12,21,24,38,44,45,49,50,52,53,55,56,57,63]. The amides contributes (amide I, amide II and amide III) are fundamental for collagen identification and for secondary structure studies [21,32,33,57]. 

The amide III band (ca. 1230–1250 cm^−1^), which resulted from C-N stretching of the peptide group, was found at 1241 cm^−1^ for T and 1243 cm^−1^ for N. The greater band intensity of T suggested the formation of more intermolecular interactions compared to N [24]. The amide II band (ca. 1540–1560 cm^−1^), which resulted from N-H bending vibration, was found at about 1541 cm^−1^ for T and 1551 cm^−1^ for N. The shift of N to a higher frequency indicated a higher content of ordered molecules compared to T [64]. The amide I (ca. 1630–1680 cm^−1^), which resulted from the stretching vibrations of the C=O bond along with polypeptide backbone or hydrogen bond coupled with the –COOH groups, was found at about 1644 cm^−1^ for T and 1652 cm^−1^ for N. The decrease of T band intensity indicated that the carbonyl groups were involved more in the cross-linking to form new bands. Thus, the amide I and II shift suggested that T had stronger hydrogen bonds and a lower degree of molecular order than N, because the shift of these peaks to higher frequencies is related to the increased hydrogen bonding in the triple helical structure [50,57].

In order to in-depth investigate collagen secondary structure, the amide I peak was further analyzed. According to the literature [38,55], five contributions were identified and analyzed in terms of peak center and relative area under the peak. The β-sheet (peak center: 1610–1642 cm^−1^), random coil (peak center: 1642–1650 cm^−1^), α-helix (peak center: 1650–1660 cm^−1^), β-turn (peak center 1660–1680 cm^−1^), and β-antiparallel (peak center 1680–1700 cm^–1^) contributes were identified and compared to the literature (Table 2). While ASC is usually found to be rich in β-sheet [53,55], PSC was revealed to have a more homogeneous distribution of contributes, with a lower β-sheet and a higher α-helix content [55]. Contributes distribution of T and N were found to have a lower β-sheet and a higher α-helix content compared to the literature data [38,53,55]. In particular, N was found to have a higher α-helix content (54%) than T (35%), suggesting its higher structural order. Additionally, according to Terzi et al. [39], the asymmetrical amide I band was de-convoluted in order to separate the contribute arising from single α-helices (ca. 1660 cm^−1^) from the one related to the triple helix organization (ca. 1630 cm^−1^) and to evaluate the relative number of triple helices for the investigated collagens. Thus, the percentage of the total amide I peak area ascribable to the triple helix peak was found to be higher for N but not statistically different from T (T: 55 ± 7 %, N: 59 ± 2%, *p* = 0.3).

### 4.4. X-rays Structural Analysis

**Figure 4:**
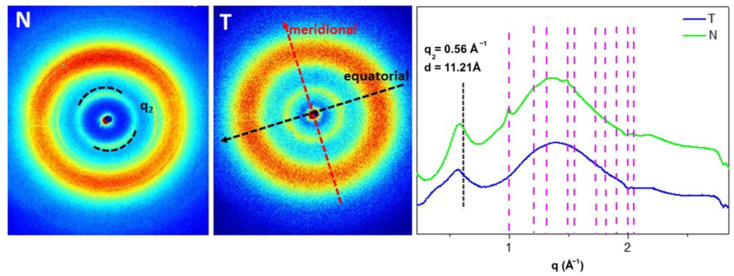
On the **left** the 2D WAXS patterns on N and T samples. The black and the red arrows show the directions along which the equatorial and the meridional diffraction signals, respectively. On the **right** the 1D diffraction profiles of both samples. The equatorial peak (q_2_) is marked with the black line. The magenta lines show the peak positions of Ultralene sachet.

As reported in the literature [32,39,65] type I collagen has a typical 2D diffraction pattern characterized by the presence of the equatorial diffraction signal q_2_ = 0.56 Å d_2_ = 11.21 Å (Figure 4, along the black arrow) ascribable to the lateral packing of collagen molecules inside the fibrillary structure, and the meridional diffraction signals q_1_ = 2.24 Å d_1_ = 2.8 Å (Figure 4, along the red arrow) related to the axial distance between amino acid residues in the triple helical conformation.

On fish skin collagen, in both T and N patterns, the meridional diffraction peak was no longer visible. In N, a preferential orientation of the equatorial signal (q_2_), marker of the molecular lateral packing retention, was clearly detectable as two small arcs with high signal intensity distributed along the same direction marked with the dashed arc, the equator (Figure 4, N). In the diffraction pattern of T (Figure 4, T), the observed equatorial q_2_ signal along the black arrow was characterized by signal intensity distributed as a continuous ring, marker of no preferential orientation, and, therefore, of a reduced packing order in the molecule. Comparing the 1D WAXS diffraction profiles, it was observed that the equatorial diffraction peaks at q_2_ ~ 0.56 ± 0.03 Å^−1^ corresponded to the average value of d-spacing of 11 Å in both samples. However, as shown in Table 3, the FWHM of the q_2_ in T is higher than in N ones, leading to a lower extent of the crystalline domain in T (37 ± 3 Å) that in N collagen (52 ± 6 Å), thus a reduced lateral packing and molecular order in T.

### 4.5. Thermal Properties

**Figure 5:**
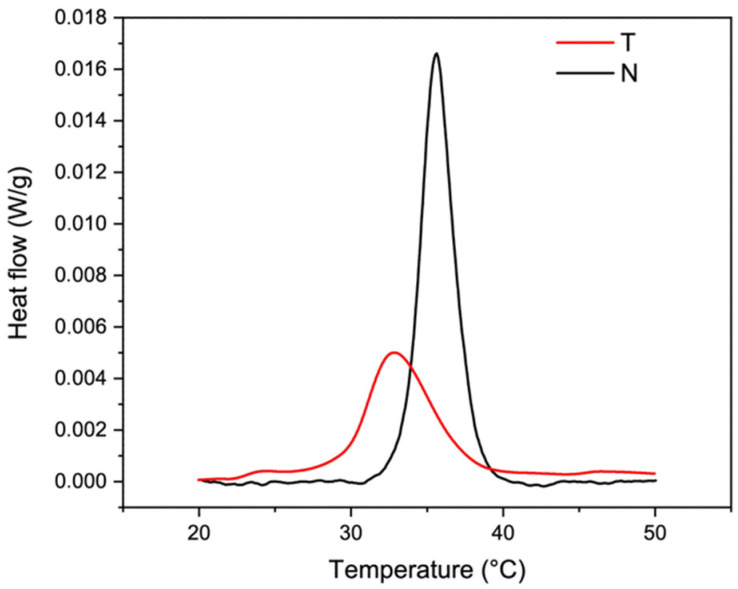
Representative DSC thermograms of T (red) and N (black) samples showing the endothermic phenomena of type I collagen denaturation.

**Figure 6:**
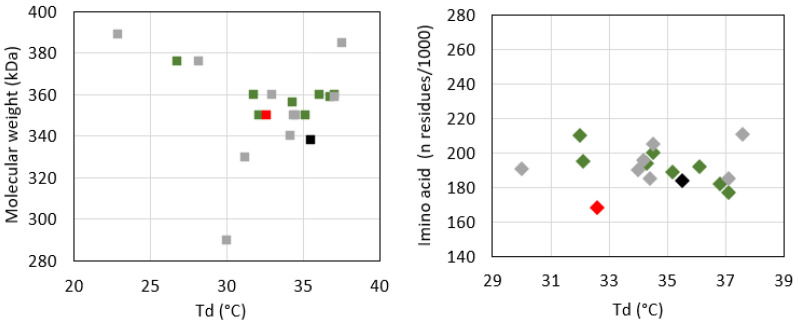
Correlation of the denaturation temperature with the hydroxyproline content (**left**) and the molecular weight (**right**), and comparison of T (red) and N (black) data with literature data on ASC (green) and PSC (grey) [12,13,22,23,24,44,45,51,52,54,56,57,58,59,66].

Representative thermograms of T and N are shown in Figure 5. While heating, the inter-chain hydrogen bonds of the right-handed superhelix break and the triple helix unfolds in random chains. The endothermal peak present within the temperature range of 0–100 °C was attributed to the T_d_ of collagen [33] that was about 32.6 ± 0.2 °C for T and of 35.5 ± 0.1 °C for N (*p* = 0.00006) [66]. According to the literature, T_d_ was found to be lower than that of land animals [9,34] and comparable to that of Tilapia skin derived collagen (Figure 6) [12,13,22,23,24,44,45,51,52,54,56,57,58,59,66]. The higher T_d_ value of N reflected its higher native structure preservation compared to T, confirmed also by the higher enthalpy necessary for the transition (T: 0.33 ± 0.05 J/g, N: 0.53 ± 0.06 J/g, *p* = 0.01) and by the higher amount of cross-linked β+γ chains (see Section 4.1) [67].

According to several studies, there is a direct correlation between the thermal stability of the proteins and their amino acid content [18,67,68]. In particular, the T_d_ is directly related to the imino acid content [22] and the non-helical region extent, which are responsible of the order and the structural compactness of the protein [59]. In this sense, the higher hydroxyproline and imino acid content of N (184 residues/1000) compared to T (168 residues/1000) might contribute to explaining its higher T_d_ value. Concerning the molecular weight, though SDS-PAGE revealed a higher molecular weight of T compared to N, this did not correspond to higher thermal resistance. Despite these experimental pieces of evidence, the analysis of the literature data did not show any clear correlation between protein molecular weight and T_d_ (Figure 6, left) on the one hand and between the protein amino acid composition and T_d_ (Figure 6, right) on the other. Reasonably, other parameters, such as growing conditions, extraction procedures (e.g., working temperature, acid treatment, pepsin-based treatment, mechanical fragmentation), and analytical techniques, may affect protein denaturation temperature [9,33,60,69,70].

### 4.6. Wettability

**Figure 7:**
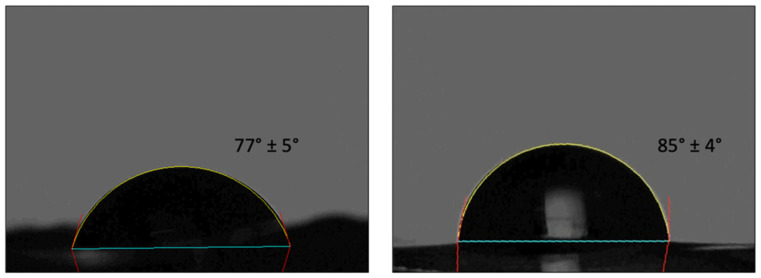
Instant contact angle on T (**left**) and N (**right**) substrates.

It is known that cell-material interactions are affected by surface properties and a moderate wettability is optimal for cell adhesion and proliferation [71]. The contact angle instantly recorded on T and N film was of 77 ± 5° and 86 ± 4°, respectively (*p* = 0.025) (Figure 7). The slightly higher hydrophilicity of T samples might contribute to different cell responses.

### 4.7. Mechanical Properties

**Figure 8:**
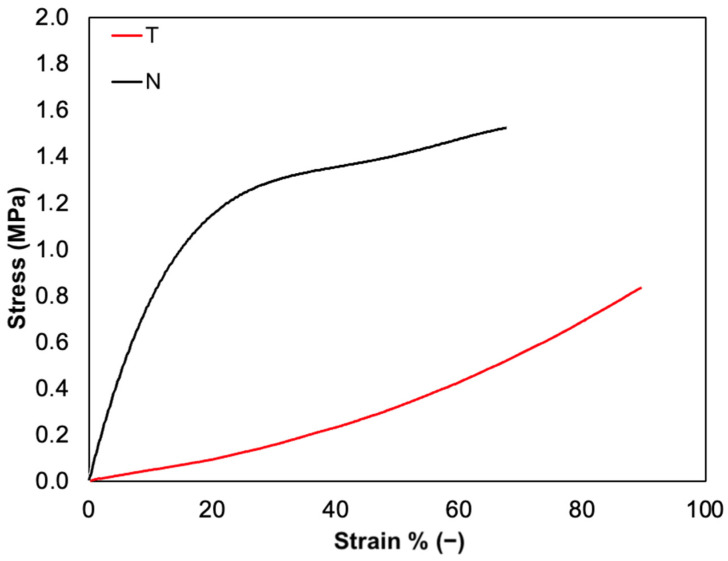
Representative stress-strain curves of T film (red) compared to N film (black).

Tensile tests were performed to investigate the mechanical properties of T and N substrates. As expected, tensile curves were characterized by a linear elastic region, followed by a non-elastic region and a rupture region (Figure 8). Although both formulations are composed only of native type I collagen, the slight differences found in their molecular structure strongly affected their mechanical behavior. The constitutive bond of T was found to be statistically different from N in terms of E, σ_max_, and ε_r_. In particular, while E and σ_max_ values of T (E_T_: 0.5 ± 0.1 MPa, σ_max,T_: 0.8 ± 0.1 MPa) were found to be about three times lower than N (E_N_: 1.6 ± 0.2 MPa, σ_max,N_: 2.5 ± 0.7 MPa) (*p* = 0.002 and *p* = 0.01, respectively), ε_r_ was found to be about 1.5 times higher than that of N (ε_r,T_: 85 ± 12 %, ε_r,N_: 59 ± 6 %, *p* = 0.02). These results suggest that the N-based substrate is characterized by higher stiffness, likely due to the broad conservation of its native structure and too strong inter-chain interactions of collagen molecules. However, a lower ε_r_ corresponds to a higher E value of N collagen. On the other hand, the T-based substrate displays greater elasticity as it reaches higher ε_r_ values but lower E. This behavior might be associated with the lower structural conservation degree that promotes the formation of inter- and intra-chain interactions between collagen molecules.

### 4.8. Biocompatibility

**Figure 9:**
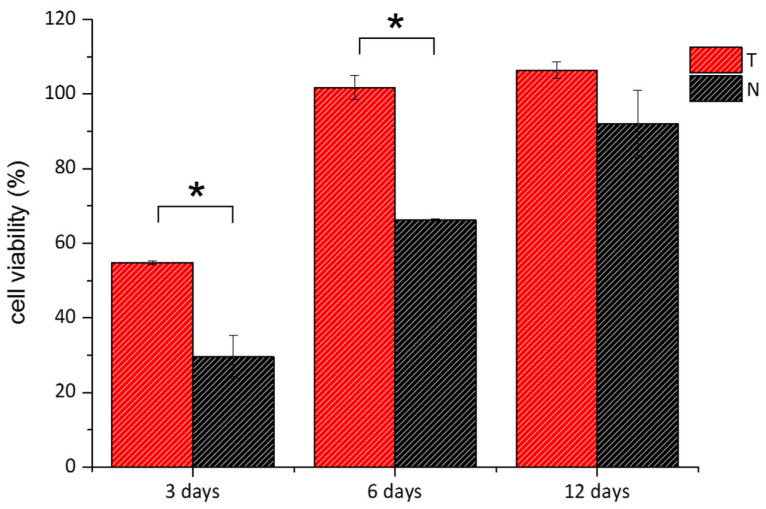
MTT viability assay of 3T3 cells grown over T (red bars) and N (black bars) was performed after 3, 6, and 12 days. Data were reported as the percentage viability of the samples over the control cells (i.e., cells grown on standard plates). (*) indicates statistical significance with *p* < 0.01.

Two cellular assays, MTT and Live/Dead, were performed to assess the cytocompatibility of the two materials. The two assays provide a collective estimation of the health status of the cells upon colorimetric evaluation of the activity of cytoplasmic and mitochondrial enzymes and of the integrity of the cell membrane. Thin collagen films were casted into culture plates, and 3T3 mouse fibroblasts were seeded over them. In both tests, cells seeded into standard multiwell plates were used as a control. Figure 9 reported cells viability percentage over time for both T and N. After 3 days, it looks that the viability of the cells grown over the two substrates was much lower than that of control cells, being equal to 54% and 29% in the case of T and N, respectively. At 6 and 12 days, the viability raised up to 100% for cells grown over the T substrates, while it reached 66% and 92% in the case of cells seeded over N collagen, respectively. From day 6, cells recovered their viability reaching values close to control samples. This cellular resilience likely indicates that the substrates do not exert a toxic effect, but rather the topography and structural features of the collagen films affect the adhesion and the replication phase of the cells, as already reported [72,73,74]. Interestingly, the differences between the metabolic response of cells grown on T and N at days 3 and 6, respectively, were statistically significant, supporting the evidence of a different affinity between materials and cells. To shed light on the interactions between the substrates and the cells, optical images of cells seeded over T, N and standard plates, respectively, were acquired (Figure S1). After 1 and 3 days, the fibroblasts grown over the T film showed the typical elongated morphology of these cells while the density was slightly lower than control samples. On the other hand, after 1 day, cells seeded on N film looked spherical, an indication of weak adhesion, while after 3 days, as cell density increased, both elongated and spherical cell clusters coexisted. This heterogeneous cell culture was maintained up to day 6, while at day 12 cell distribution was similar to that of the control.

**Figure 10:**
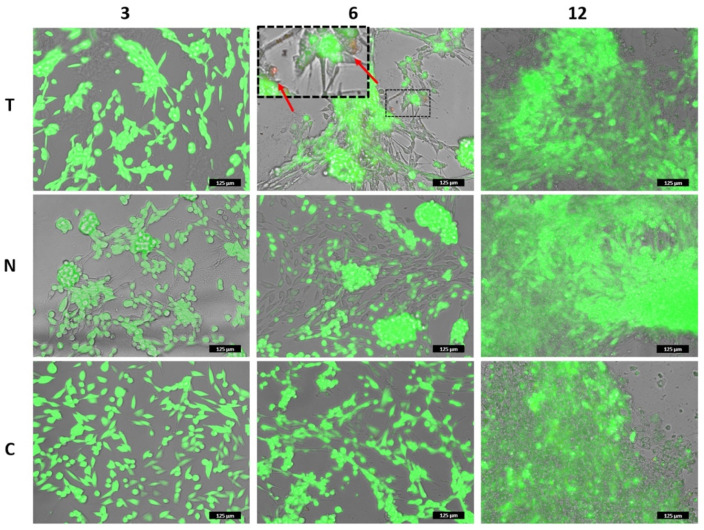
Live/Dead assay of 3T3 cells grown over T and N was performed after 3, 6, and 12 days. The images correspond to the overlapped fluorescent channels (green for calcein signal and red for ethidium homodimer) and the bright field. The inset in the upper-central panel shows some dead cells among the live ones. Note: in the panels in which cells are localized on different focal lanes (elongated on the bottom plane and clustered on the upper plane), the green fluorescent channel has been slightly reduced to allow the detection of the dead cells. Please, refer to the images of Figures S2–S4 for the original signal intensity for each fluorescent channel. Scale bar is 125 µm.

Live/Dead assay was performed under the same experimental conditions. As shown in Figure 10, fibroblasts looked viable in both samples and at all the imaged times (Figures S2–S4). Few dead cells were detected, as reported in the inset of Figure 10. The morphology and the distribution of the cells were in agreement with the previous images and confirmed that the results of the MTT assay did not indicate inherent toxicity of the biomaterials but a delayed replication time as compared to the control.

**Figure 11:**
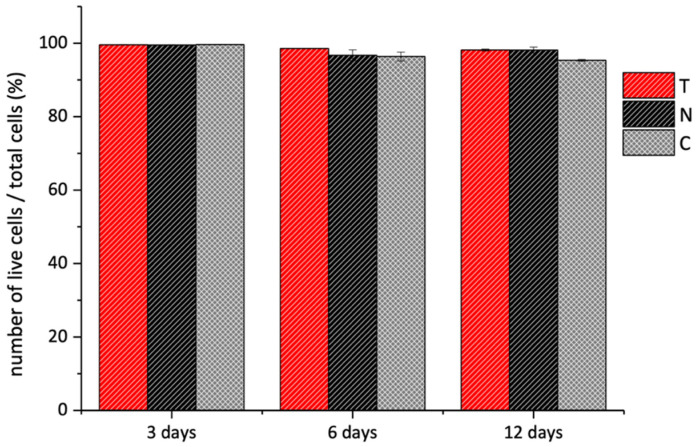
Live/Dead assay of 3T3 cells grown over T (red bars) and N (black bars) substrates performed after 3, 6, and 12 days. The data are presented as the average percentage of live cells over the number of total cells. The analysis was performed through Image J Software over three different fluorescent images for each sample and measuring the green (i.e., live cells) and red (i.e., dead cells) pixels of each image.

The quantitative analysis of the fluorescent pixels by the Image J Software 1.52t (NIH, USA) allowed us to estimate the number of living cells. The histograms in Figure 11 showed that the percentage number of viable cells over the total cells was very similar in all the samples. Based on these findings, we concluded that the T and N were biocompatible but presented different surface topography that affected cellular adhesion and replication time.

## 5. Discussion

Collagen is the most abundant structural protein of animal tissues that provides strength and structural stability on the one hand and performs highly specialized regulatory functions on the other. The interesting structural and biological properties of collagen, besides its biodegradability, high biocompatibility, and low immunogenicity [2,3,4,5,6,7], made it one of the most widely required biomaterials for food and healthcare applications, including medical care, pharmaceutics, and cosmetics [75].

The increasing demand for collagen brought to the examination of several extraction sources, including mammals (i.e., bovine, swine, equine, ovine, rodents), birds, and marine organisms (i.e., fish and invertebrates), with the aim of finding the optimal one in term of biocompatibility, safety, and availability [9]. The best extraction source is represented by mammals for the high sequence homology with human collagen [3]. However, mammals-derived collagen use is limited because of issues such as the high immune response rate, zoonosis transmission risks, and religious/cultural constraints [3,8,9]. Due to these restrictions, alternative safer sources were investigated. 

In this perspective, marine organisms seemed to be free from all the aforementioned limitations [9,13] and attracted interest as safe and abundant sources for collagen extraction [9,11,12]. In particular, among fish species, Nile tilapia (*Oreochromis niloticus*) and its byproducts (i.e., skin, scale, bone) are emerging as alternative sources for collagen isolation. Being one of the World’s most representative species of the fisheries and aquaculture food sector for its fast growth speed, adaptability, reproducibility in captivity, easy feed, and easy processing [22,25,26] it offers a huge quantity of byproducts (70–85% of the total weight of catch) that could be exploited to develop waste recovery technologies to obtain high added-value products and at the same time to reduce the environmental pollution related to their breeding and processing [10,19,20]. Additionally, eco-sustainable aquaponic systems give the possibility to obtain hazardous-free Tilapia biomass with an additional added value and controlled properties over time. Thus, the possibility to valorize the huge quantity of fish industry waste and byproducts as collagen source makes Tilapia-derived collagen eco-friendlier and particularly attractive in terms of profitability and cost-effectiveness. 

In this work, type I collagen extracted according to a proprietary process from the skin of aquaponics-derived Tilapia was characterized from a chemical, physical, mechanical, and biological point of view and compared with a commercially available isoform. Particular attention was paid to its native structure preservation since it is widely known that it not only influences bioengineering parameters but also cell-biomaterial interaction and thus cell processes [65,76,77,78].

First of all, the protein integrity and the presence of other protein species were evaluated. The revealed electrophoretic pattern confirmed the purity of the extracts and the extraction process accuracy since there were only two bands visible of about 120 kDa and 110 kDa attributable to α1(I) and α2(I) chain of type I collagen [23,45,46,52,54,55,56]. In particular, T was found to have a molecular weight slightly higher than N (T: α1 = 120 ± 3 kDa, α2 = 110 ± 2 kDa; N: α1 = 116 ± 2 kDa, α2 = 106 ± 1 kDa) and a content of high molecular weight components lower than N (T: 0.28 ± 0.11; N: 0.77 ± 0.16). The conversion of more cross-linked chains (β, γ) into non-cross-linked monomer chains (α1, α2) directly indicated a greater efficacy of T extraction method in disassembling fibril units [23].

The integrity of T secondary structure and its ultrastructure was evaluated by means of FT-IR and WAXS. The presence of the three amide contributes (amide I: 1644 cm^−1^ for T, 1652 cm^−1^ for N; amide II: 1541 cm^−1^ for T, 1551 cm^−1^ for N; amide III: 1241 cm^−1^ for T, 1243 cm^−1^ for N) allowed to confirm the protein identity, that was attributable to type I collagen, comparable to N and according to literature data about Tilapia skin derived collagen [12,21,24,38,44,45,49,50,52,53,55,56,57,63]. The amides shift suggested that T had a lower degree of molecular order than N, besides a lower α-helix content (T: 35%; N: 54%). However, from the deconvolution of the amide I came out a slightly lower but not statistically different triple helix content of T from N (T: 55 ± 7%, N: 59 ± 2%), suggesting the employment of less tightened extraction conditions and better preservation of native collagen structure of N. Higher partial preservation of the native lateral packing of collagen molecules in N with respect to T was observed through WAXs measurements. However, the presence of a clear equatorial signal in T collagen allowed us to identify the presence of native structure, although it is less organized than the N one. Indeed, the continuous ring intensity distribution of the equatorial diffraction signal in T, associated with the decrease of the extension of the crystalline domain, obtained by the peak analyses, is a clear marker of the presence of lateral packing arrangement of triple helices. While in N collagen triple helices are arranged by a preferential orientation in a larger crystalline domain.

In order to ascribe protein structural differences to compositional variations, protein amino acid composition was also investigated. The amino acid composition of T was found to be comparable to N, and the literature data on Tilapia skin collagen [22,23,24,44,45,50,51,52,54,57,58,59], common aquatic organisms [3,9], and to be very similar to land mammals [3]. Thus, both T and N were found to be rich in glycine (T: 325/1000 residues; N: 345/1000 residues), proline (T: 112/1000 residues; N: 127/1000 residues), alanine (T: 123/1000 residues; N: 142/1000 residues), and hydroxyproline (T: 56/1000 residues; N: 57/1000 residues). The imino acid content of T (168/1000 residues) and N (184/1000 residues) revealed a greater N structural rigidity since a higher pyrrolidine rings content imposes more restrictions on the polypeptide chain conformation.

To investigate how the collagen isolation procedure affects its thermal stability, thermal behavior was investigated. Thermograms revealed a T_d_ value of T (32.6 ± 0.2 °C) that was similar to that one of N (35.5 ± 0.1 °C) and according to literature data about Tilapia skin-derived collagen [12,13,22,23,24,44,45,51,52,54,56,57,58,59,66]. In addition, the lower denaturation enthalpy value of T compared to N (T: 0.33 ± 0.05 J/g; N: 0.53 ± 0.06 J/g) indicated that lower energy is required to start the protein unfolding process in T collagen, likely due to slightly lower preservation of the native structure than N. This result is accordance with the SDS-PAGE, FT-IR and WAXS findings. According to several studies, there is a direct correlation between protein thermal stability and their imino acid content [18,67,68] and non-helical region extent [59]. While the imino acid content might contribute to explain protein T_d_ value, any correlation with protein molecular weight was found. Additionally, literature data analysis did not show any clear correlation between protein T_d_, amino acid composition, and molecular weight. Reasonably, other parameters, such as growing conditions, extraction procedures, and analytical techniques, may strongly affect protein features [9,33,69] and deeply affect data comparative analysis. Anyhow, T_d_ is key parameter to validate Tilapia-derived collagen as a raw biomaterial for healthcare applications, and its lower value than that of land animals [9,34] restricted its use. Nevertheless, various strategies have been developed for improving fish collagen thermal stability. Among these, recalling to chemical cross-linking (e.g., carbodiimides, genipine) [79] allowed to successfully increase fish collagen T_d_ up to body temperature and thus to show great promise for biomedical or clinical use.

Cellular studies indicate that T substrates are highly biocompatible and support the adhesion, growth, and spreading of fibroblasts. In addition, the topography of the substrate looks to play a crucial role in determining the interactions among the substrate and the cells. The comparison of the two collagen substrates shows that adhesion of the fibroblasts over T occurs faster than over N rapidly. On the other hand, the tensile tests showed that T substrates are softer than N, and several literature studies evidenced that fibroblasts generally prefer stiffer surfaces [80,81]. Interestingly, the experimental evidence suggests that beyond surface stiffness, the structure and the organization of collagen-based substrates affect the interaction with the cells.

## 6. Conclusions

Type I collagen is one of the most widely used biomaterials for healthcare applications for its well-known advantages. Its increasing demand brought to the examination of several extraction sources, with the aim of finding the optimal one in term of biocompatibility, safety, and availability. Although the discovery of numerous alternatives, the best extraction source is currently represented by mammals. However, several issues limited their use. In this circumstance, marine organisms seemed to be free from many limitations and promising raw materials sources for collagen extraction. Among fish species, Nile tilapia byproducts emerged as an alternative source for collagen isolation because of their abundance from fisheries and aquaculture plants. The recovery from eco-sustainable aquaponic systems gave the possibility to obtain hazardous-free Tilapia biomass with additional added value and controlled properties.

In this perspective, type I collagen was characterized from a chemical, physical, mechanical, and biological point of view and compared with a commercially available isoform. Particular attention was paid to its native structure preservation since it is widely known that it influences not only bioengineering parameters but also cell-biomaterial interaction and thus cell processes. All analyses confirmed that the proprietary process optimized for type I collagen extraction allowed to isolate pure native collagen and preserve its native conformational structure. The slight differences observed between T collagen and the commercial preparation may be due to different breeding conditions, besides extraction and purification procedures, resulting in collagen molecular organization alteration and thus in its bioactivity modification. These evidence, in combination with the positive feedback of the cellular studies, suggested that type I collagen from aquaponics-raised Tilapia skin could be a suitable high-controlled alternative biomaterial for healthcare application. The bioactivity of fish collagen makes its use in multiple forms (including native, gelatin, and peptide form) in rapid expansion for specific biomedical pharmaceutical, cosmetics, nutraceutical, nutricosmetic, and food applications. Although fish collagen has been proposed as an alternative biomaterial for tissue engineering applications, there is a lack of literature data about its immunogenicity. The host immunologic response is a critical aspect when considering it for clinical implementation since the success of a biomaterial-based formulation for healthcare applications is directly related to the immune response to the selected material. Based on these findings, further studies should be performed to evaluate the immunogenicity response triggered by the isolated type I collagen. Additionally, to the best of our knowledge, no studies were available on the effective lower cost of fish-derived collagen compared to mammalian-derived collagen, an accurate cost-effectiveness study should be performed.

## Figures and Tables

**Table 1:** Amino acid composition of T and N and comparison with the literature data on type I collagen (ASC and PSC) isolated from Tilapia skin [22,23,24,44,45,50,51,52,54,57,58,59]. Results are expressed as residues/1000 total amino acid residues.

Amino Acids	T	N	ASC									PSC							
			[22]	[54]	[50]	[23]	[24]	[52]	[45]	[58]	[59]	[59]	[23]	[51]	[24]	[52]	[57]	[45]	[44]
Alanine	123	142	119	122	141	104	126	171	87	98	123	127	104	118	124	180	124	85	98
Glycine	325	345	356	333	287	252	338	230	322	332	354	360	256	319	333	230	343	343	216
Valine	20	18	17	26	33	19	21	20	22	26	19	18	17	16	19	20	18	25	23
Leucine	23	19	20	26	24	27	24	20	22	22	23	22	23	2	23	20	21	20	28
Isoleucine	10	8	8	16	11	13	10	10	11	7	9	9	9	8	10	10	9	16	14
Proline	112	127	128	115	127	110	110	120	106	112	132	136	111	113	119	121	125	115	122
Methionine	2	1	5	23	14	14	3	8	9	34	10	9	15	6	2	7	6	10	4
Serine	32	32	32	32	19	31	35	21	31	32	35	35	26	33	35	20	31	28	32
Threonine	18	25	22	24	27	26	23	20	15	24	25	25	28	24	23	20	23	17	23
Phenylalanine	16	11	13	20	18	18	14	11	10	16	13	12	12	12	14	11	12	16	23
Aspartate	61	56	42	44	56	60	47	50	40	47	45	45	60	41	46	50	42	37	51
Glutamate	72	69	69	67	51	96	78	91	98	71	71	69	97	68	76	91	70	93	93
Lysine	29	28	20	26	52	35	24	41	32	28	25	25	37	24	23	31	23	22	34
Histidine	1	0	6	3	14	8	8	10	10	7	6	5	6	5	8	10	6	12	12
Tyrosine	4	1	3	3	5	5	4	3	9	1	4	2	2	2	3	4	3	6	6
Cysteine	0	0	0	4	1	0	2	0	0	2	0	0	0	0	3	0	0	0	9
Hydroxyproline	56	57	82	62	70	84	79	80	86	83	50	49	85	77	86	70	86	70	134
Arginine	27	35	58	51	41	82	55	40	90	52	53	52	96	52	51	40	58	85	78
Imino acids	168	184	210	177	197	194	189	200	192	195	182	185	196	190	205	191	211	185	256
Tot.	1000	1000	1000	1000	1000	1000	1000	1000	1000	1000	1000	1000	1000	1000	1000	1000	1000	1000	1000

**Table 2:** Secondary structure percentage (%) analysis of T and N in the 1600–1700 cm^−1^ spectral range in comparison with the literature data [38,53,55].

Collagen	β-Sheet 1610–1642 cm^−1^	Random Coil 1642–1650 cm^−1^	α-Helix 1650–1660 cm^−1^	β-Turn 1660–1680 cm^−1^	β-Antiparallel 1680–1700 cm^−1^
T	7	36	35	16	5
N	4	19	54	19	4
[55] ASC	30	25	24	21	-
[55] PSC	26	26	30	17	-
[38] ASC	24	14	20	33	9
[53] ASC	52	16	14	13	5

**Table 3:** Mean values of lateral packing (q_2_) of collagen molecules in the samples in the reciprocal space (Å^−1^) and the corresponding values in the direct space (Å). The FWHM value of the analyzed diffraction peaks is also shown, and the relative value of the crystalline domain for the lateral packing. The mean values were obtained from the mean of the experimental values measured for each sample.

Sample	Reciprocal Space (Å^−1^)		Direct Space (Å)	
	q_2_ Equatorial	FWHM	d_2_ Equatorial	Cristalline Domain
T	0.55 ± 0.03	0.169 ± 0.002	11	37 ± 3
N	0.57 ± 0.03	0.120 ± 0.001	11	52 ± 6

## Supplementary Materials

The following supporting information can be downloaded at: https://www.mdpi.com/article/10.3390/polym14091865/s1, Figure S1: optical images acquired after 1-, 3-, 6-, and 12-days growth of 3T3 cells over T, N substrates and standard plates -control samples- (C). Figure S2. Live/dead assay of 3T3 cells performed after 3 days growth over T and N substrates. Control samples are reported as well (C). The first column refers to the overlaid channels, while the others correspond to the bright field, to the green channel of calcein and to the red channel of ethidium homodimer, respectively. Figure S3. Live/dead assay of 3T3 cells performed after 6 days growth over T and N substrates. Control samples are reported as well (C). The first column refers to the overlaid channels, while the others correspond to the bright field, to the green channel of calcein and to the red channel of ethidium homodimer, respectively. Figure S4. Live/dead assay of 3T3 cells performed after 12 days growth over T and N substrates. Control samples are reported as well (C). The first column refers to the overlaid channels, while the others correspond to the bright field, to the green channel of calcein and to the red channel of ethidium homodimer, respectively.
