# Supplementary material for: Aquaponics-Derived Tilapia Skin Collagen for Biomaterials Development
Source: Polymers (Basel). 2022 May 2;14(9):1865. doi: 10.3390/polym14091865 (PMC9103308; doi:10.3390/polym14091865)
Supplement: Supplementary file 1 [file polymers-14-01865-s001.zip › polymers-1680885-SI.pdf]

polymers

Supplementary materials

Aquaponics-Derived Tilapia Skin Collagen for Biomaterials Development

Nunzia Gallo[1,*], Maria Lucia Natali[1,2], Alessandra Quarta[3], Antonio Gaballo[3], Alberta Terzi[4], Teresa Sibillano[4], Cinzia Giannini[4], Giuseppe E. De Benedetto[5], Paola Lunetti[6], Loredana Capobianco[6], Federica Stella Blasi[1], Alessandro Sicuro[6], Angelo Corallo[1], Alessandro Sannino[1], Luca Salvatore[1,2]

[1] Department of Engineering for Innovation, University of Salento, Via Monteroni, 73100 Lecce, Italy;
[2] Typeone Biomaterials Srl, Via Vittorio Veneto, 73036 Muro Leccese (LE), Italy
[3] CNR Nanotec, Institute of Nanotechnology, Via Monteroni, 73100 Lecce, Italy
[4] Institute of Crystallography, National Research Council, 70125 Bari, Italy
[5] Department of Cultural Heritage, University of Salento, Via Monteroni, 73100 Lecce, Italy
[6] Department of Biological and Environmental Sciences and Technologies, University of Salento, Via Monteroni, 73100 Lecce, Italy
* Correspondence: nunzia.gallo@unisalento.it

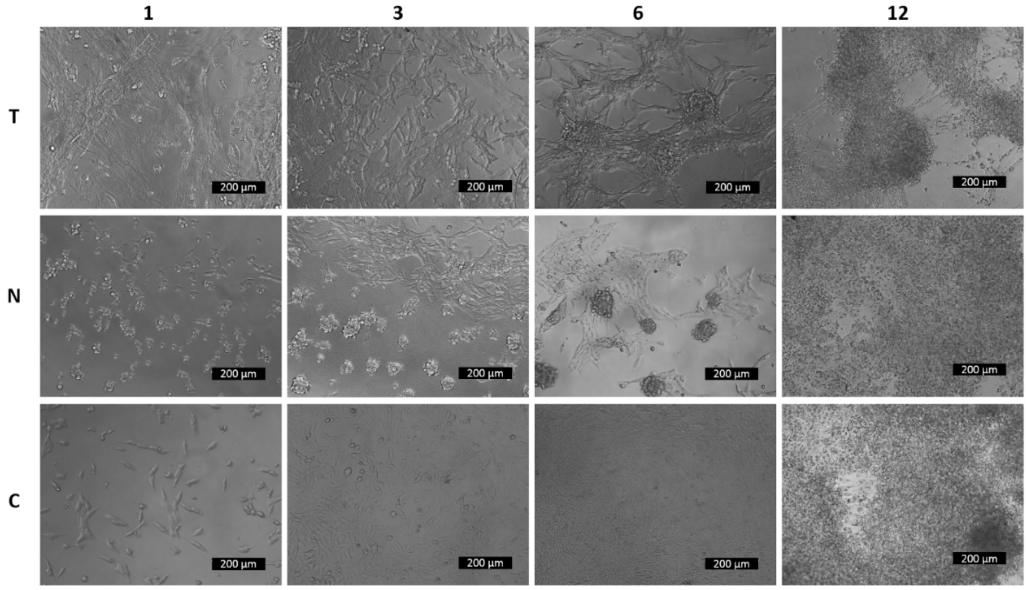

Figure S1. Optical images acquired after 1, 3, 6 and 12 days growth of 3T3 cells over T, N substrates and standard plates -control samples- (C).

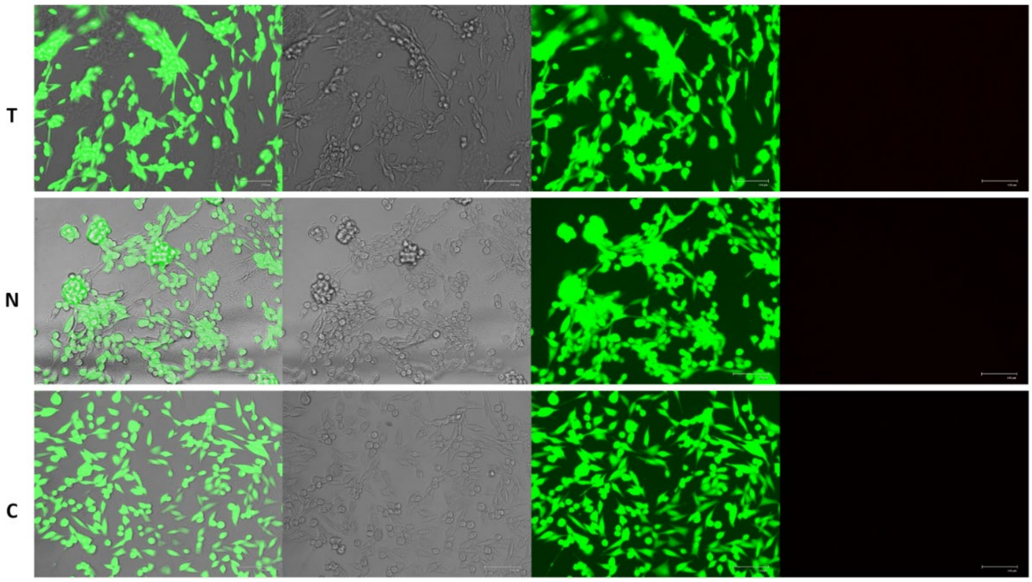

Figure S2. Live/dead assay of 3T3 cells performed after 3 days growth over T and N substrates. Control samples are reported as well (C). The first column refers to the overlaid channels, while the others correspond to the bright field, to the green channel of calcein and to the red channel of ethidium homodimer, respectively.

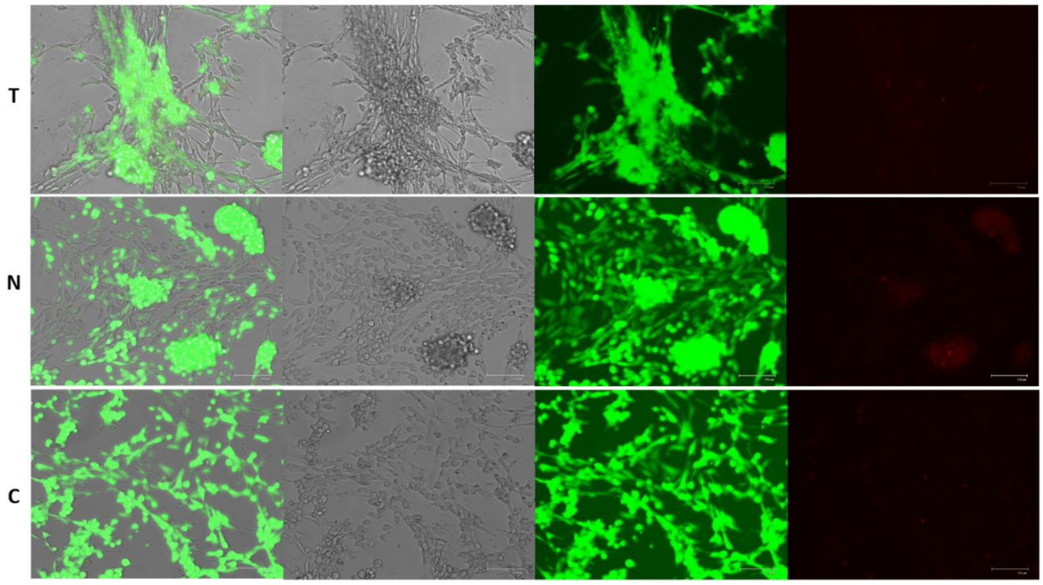

Figure S3. Live/dead assay of 3T3 cells performed after 6 days growth over T and N substrates. Control samples are reported as well (C). The first column refers to the overlaid channels, while the others correspond to the bright field, to the green channel of calcein and to the red channel of ethidium homodimer, respectively.

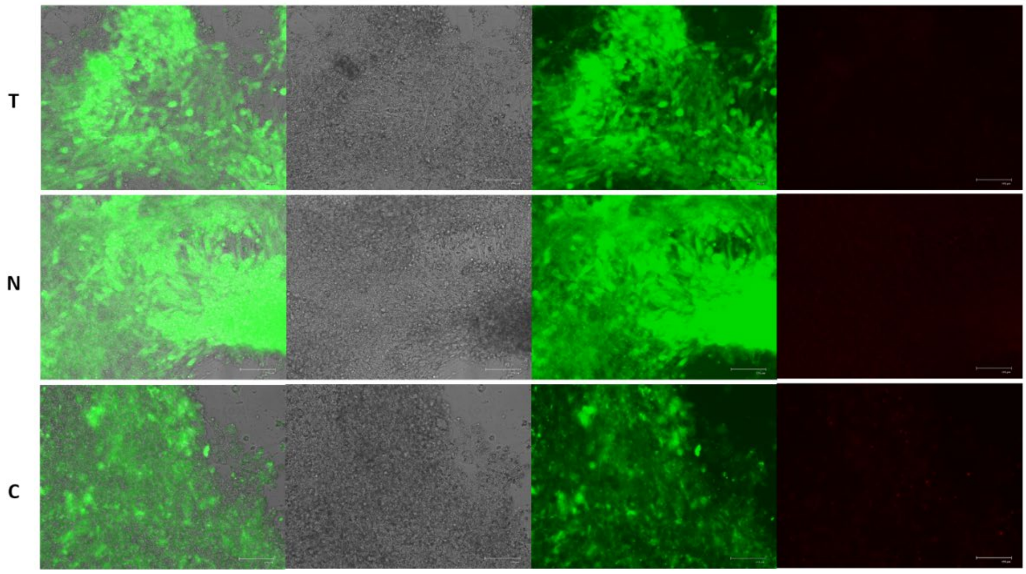

Figure S4. Live/dead assay of 3T3 cells performed after 12 days growth over T and N substrates. Control samples are reported as well (C). The first column refers to the overlaid channels, while the others correspond to the bright field, to the green channel of calcein and to the red channel of ethidium homodimer, respectively.
